# Characterizing the demographics of chronic pain patients in the state of Maine using the Maine all payer claims database

**DOI:** 10.1186/s12889-018-5673-5

**Published:** 2018-06-28

**Authors:** Jennifer Malon, Parth Shah, Woon Yuen Koh, Gary Cattabriga, Edward Li, Ling Cao

**Affiliations:** 1 Center for Excellence in the Neurosciences, University of New England, 11 Hills Beach Rd., Biddeford, ME 04005 USA; 2 College of Osteopathic Medicine, University of New England, Biddeford, ME USA; 3 Department of Mathematical Sciences, University of New England, Biddeford, ME USA; 4 Center for Community and Public Health, Portland, ME USA; 5 College of Pharmacy, University of New England, Portland, ME USA

**Keywords:** Chronic pain, Maine, Opioid, Maine all payer claims database (MEAPCD), ICD-9 code

## Abstract

**Background:**

Chronic pain is currently a significant health problem in the United States. A comprehensive strategy is needed to increase prevention of chronic pain and to improve care for chronic pain patients. However, development of a successful strategy relies, in part, on a better understanding of the demographics and socioeconomics of patients living with chronic pain conditions. The current study was designed to understand the burden of chronic pain in the state of Maine by identifying the prevalence of chronic pain and its relationship with selected demographic and socioeconomic factors in Maine.

**Methods:**

The Maine All Payer Claims Database (MEAPCD) (2006**–**2011) was used in the secondary data analysis to assess the demographic characteristics (such as age, sex, insurance type, and county of residence) of chronic pain patients in Maine. Chronic pain patients were identified based on the presence of pre-identified chronic pain-associated ICD-9 code(s) and opioid prescription information. Potential associations between the prevalence of chronic pain and a number of socioeconomic factors were determined by comparisons to Maine Census data.

**Results:**

More women in the state were identified as having chronic pain across all counties and all age groups (> 10 years old). Surprisingly, the majority of chronic pain patients were identified based on the diagnostic code criteria and not the opioid prescription criteria. A greater utilization of public health insurance was seen within the chronic pain patients. At the county level, although neither education level nor income were associated with the prevalence of chronic pain, these factors significantly correlated with the usage of public health insurance.

**Conclusions:**

Further detailed characterization of the chronic pain patient population in the state of Maine, using multiple data sources, can help design population-targeted strategies to prevent and manage chronic pain.

**Electronic supplementary material:**

The online version of this article (10.1186/s12889-018-5673-5) contains supplementary material, which is available to authorized users.

## Background

Chronic pain, which can range from mild to severe, is defined as pain that persists or progresses beyond the ordinary duration of time required for the body to heal from an insult or injury [1]. Reports approximate that the number of adult chronic pain sufferers in the United States is around 100 million [1, 2]. The National Institutes of Health, along with the Institute of Medicine, have stated that: “Pain is a significant public health problem in the United States” [1]. For this reason, National Pain Strategy was developed to emphasize the need for the development of a comprehensive population-based strategy to address the public health problem, improve prevention and care for chronic pain patients, address the health disparities associated with the disease, improve public awareness around chronic pain conditions, improve service and delivery of care to patients, and increase professional education and training. Furthermore, optimal solutions for treating chronic pain are urgently needed as chronic pain-associated annual costs are estimated to range from $560 to $635 billion [2]. These costs are more complex than just the cost of medication and doctor office visits. Costs also include the loss of productivity in work, and disability assistance needed until a person suffering from chronic pain is able to return to work, if able to return to work at all [3–5]. Altogether, these facts point to the need for an increased understanding of the demographics and socioeconomics of patients living with chronic pain.

The public health problem surrounding chronic pain is further complicated by the use of opioids as a treatment for chronic pain. Opioids have been the go-to treatment for chronic pain for the past 30 years, despite the lack of studies to back up the efficacy or safety of these drugs for treatment of chronic pain [6–10]. Recently, 2.1 million Americans were reported to be addicted to prescription opioids, and six out of ten drug overdose deaths involved opioid use [11]. Nationally in 2014, there were 18,893 opioid-related deaths, which is a 3.4 increase since 2001 [12]. Now officially classified as an epidemic, the current opioid crisis has been fueled by the overprescribing of opioids, lack of adequate comprehensive treatment of pain, and limited understanding of the etiology of chronic pain. Epidemiological data has shown that use of opioids results in greater likelihood of patients developing an addiction to opioids [13–15]. Pain patients that have previously been prescribed opioids are more susceptible to substance abuse disorders [16]; one study estimated a 25 times lower rate of abuse and/or addiction in patients without a prior history of opioid use compared to patients with prior use (0.19% vs. 5%, respectively) [17]. While opioids are still an effective treatment for some acute and chronic pain sufferers, patients treated with opioids often develop tolerance to opioids that leads to the subsequent increases in opioid dosages for pain management. Thus, the use of opioids in treatment of chronic pain remains a gray area in medicine and the efficacy and safety of an opioid prescription needs to be determined on a case-by-case basis. Furthermore, chronic pain has been increasingly recognized as a biopsychosocial condition [18–20] and comprehensive interdisciplinary management of chronic pain is preferred over medication alone [21].

According to the US Census, Maine was the oldest state in the country in 2010 [22], and the second oldest in 2015 [23]. In 2010, 15.9% of Maine residents were over age 65 [24], and the median age of Maine residents was 42.7 [24], which was greater than the US median age of 37.2 [25]. While the age range with the greatest numbers of chronic pain sufferers is early adulthood to middle age [26–28], the numbers of chronic pain sufferers relative to the total population of a given age increases with age [29, 30]. Therefore, with its significantly larger older population, Maine is more likely to have a greater chronic pain population and face an increase in chronic pain-associated healthcare burden. Also of significance is the fact that Maine is one of the states that has been hit hard by the opioid epidemic. For example, in 2015, there were 272 deaths in Maine from drug misuse; 111 of those deaths were related to use of opioids (or 41%), an increase from 104 in the previous year [31].

In this study, to address the knowledge gap regarding the characteristics of the chronic pain population in the state of Maine and the goals of the National Pain Strategy, we identified and characterized the population of Maine residents who suffer from chronic pain through a secondary database analysis using the Maine All Payer Claims Database (MEAPCD) (Data collected between 2006 and 2011). The relationship between the prevalence of chronic pain and a series of social and economic factors were also assessed in combination with the Maine 2010 Census data. We expect that our current study will be the beginning of a series of studies that will help to 1) identify the common demographic, socioeconomic, and geographic factors associated with the chronic pain population within the state of Maine, 2) estimate the burden of chronic pain imposed on the Maine economy through public healthcare needs, and 3) bring awareness to this public health issue and further reduce stigma towards chronic pain patients. All of these factors are vital pieces of the puzzle that need to be put together to create a more efficient understanding of chronic pain conditions and better management of chronic pain both at the individual patient level and at the state level.

## Methods

### Databases

The MEAPCD (Medical and Pharmacy) collected for the time period between the years 2006 and 2011 was used to identify patients with chronic pain. Individuals were identified if they met one of the two criteria below based on the report by Tian et al. 2013 [28], in which the combination of ICD-9 codes and opioid prescription has a positive predictive value (PPV) of 98% in identifying chronic pain patients:

1. Individual possesses ICD-9 code that is ‘highly’ likely to indicate chronic pain (338.Xx) with one diagnosis per calendar year, **or** an ICD-9 code ‘likely’ to indicate chronic pain (non-338.Xx code) with two diagnoses per year separated by at least 30 days within the calendar year. (refer to Tian [28] for specific codes.)
  **Exclusions:** Similar to the study by Tian, due to the significant differences in management, individuals that had diagnoses for migraine or other headache, facial pain, and cancer pain (338.3×) while without the above included diagnosis codes were excluded.
2. Daily use of one or more opioid drugs for at least 90 consecutive days within the calendar year. See Additional file 1 for a list of all opioid drugs that were prescribed by providers in the state of Maine during the selected study time period (2006–2011).

From the identified data set, age, gender, and insurance type were extracted and analyzed to characterize the demographics of chronic pain patients in the state of Maine. When referring to geographic area, all data is reported at the state and county levels.

### Census data

For 2010, chronic pain demographics were compared to Maine census data (Census.gov) [24]. From the census data, age, gender, education level, income, type of insurance, and employment status were collected at the state and county levels.

### Statistics and data interpretation

Chronic pain patients were identified according to the definition described above and reported for individual years between 2006 and 2011. During the analysis, we discovered that data from 2011 were not complete and data from 2008 could not be extracted correctly. Thus, only data from 2006, 2007, 2009, and 2010 were used for further analysis. For 2010, the identified cohort was divided by the corresponding total population (from the Maine census data) to determine the prevalence of chronic pain within specific populations. Demographics are reported in aggregated format. Furthermore, the prevalence of chronic pain within each county was compared to education level, income, and employment status within each county using data reported by the Maine Census. The two-sample z test for the difference between proportions was performed to determine differences in the percentages of chronic patients within the population between genders. The one-sample z test for proportions was performed to determine overall differences in insurance utilization, and differences in insurance utilization by gender. The t test for correlation coefficient was performed to determine the significance of the correlation coefficient between pairs of variables.

## Results

### Chronic pain cohorts

**Fig. 1:**
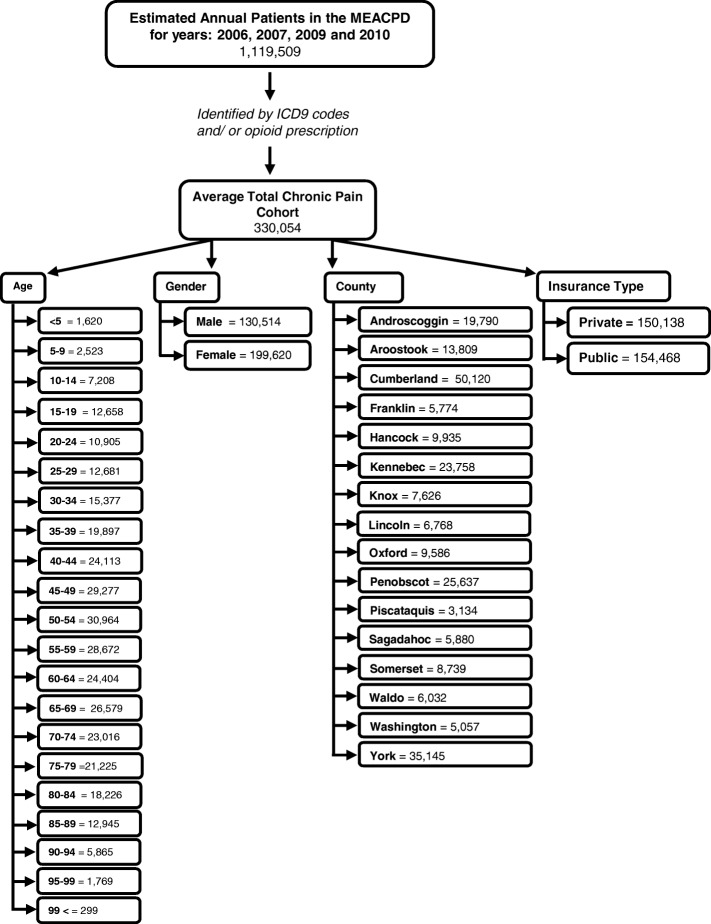
Demographics of identified chronic pain population. The chronic pain cohort was identified within the total Maine All Payer Claims Database for the years 2006, 2007, 2009, and 2010, respectively using the pre-determined ICD-9 codes and opioid prescription criteria (see text for detail). Data from each year were broken down into age, gender, insurance type, and residing county of chronic pain patient, and then analyzed. The chart shows the average of the four years for each of the indicated demographic factors

The chronic pain cohort was identified within the total population collected in the MEAPCD for the years 2006, 2007, 2009, and 2010, as described in the Methods section. Over these four years, the average total population per year within the database was 1,119,509, which reflects the total Maine population (1,328,361 as reported in the 2010 census) minus the uninsured Maine population (89.87% as reported in the 2010 census) and ± 5% error of the database [24]. The annual average of the identified chronic pain cohort was 330,054 people, with 278,059 identified in 2006, 341,491 in 2007, 363,789 in 2009, and 336,878 in 2010. Interestingly, the overall number of chronic pain patients identified in 2006 was much less than subsequent years (for years 2007, 2009, and 2010, overall numbers of identified chronic pain patients were similar among years), yet the population of the state was steady during the years included in the study, with an annual increase of 0.03–0.04% [24]. Figure 1 shows the demographic information collected from the database regarding the identified chronic pain population, including age distribution, gender, county of residence, and types of insurance used.

**Fig. 2:**
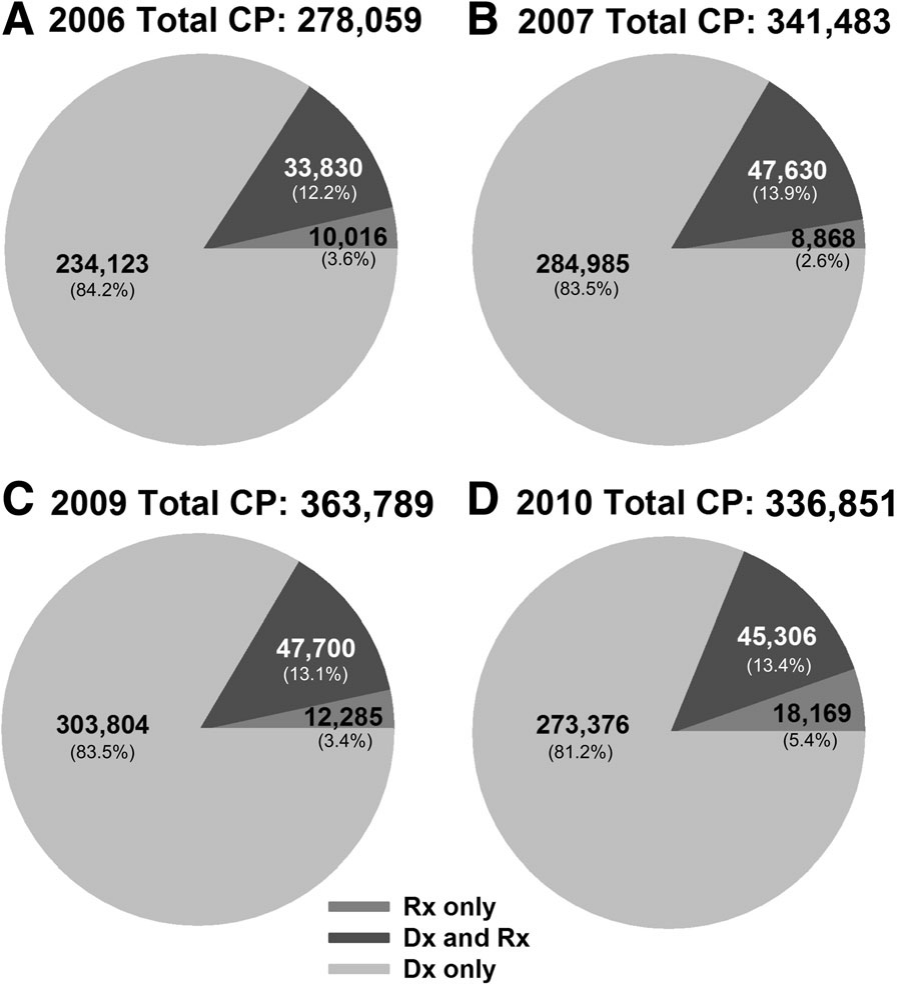
Distribution of cohort identification criteria. Pie charts represent the total number and percent (within parentheses) of patients who met the ICD-9 code parameters, opioid parameters, or ICD-9 code plus opioid parameters, as described in the Methods section. Parameters are reported for years 2006 (**a**), 2007 (**b**), 2009 (**c**), and 2010 (**d**)

For all four years, the majority (above 80%) of chronic pain patients were identified by chronic pain-related ICD-9 codes (Fig. 2, light gray). While some individuals (12.2–13.9%) were identified by both criteria (Fig. 2, dark gray), only a smaller number of patients (2.6–5.4%) were identified by opioid prescriptions criteria alone. However, the overall number of individuals who met the opioid prescription criteria in our cohort increased every year (43,846 (15.8%) in 2006, 56,498 (16.5%) in 2007, 59,985 (16.5%) in 2009, and 63,475 (18.8%) in 2010) with the largest increase occurring in 2010.

### Age and gender of chronic pain cohorts

**Fig. 3:**
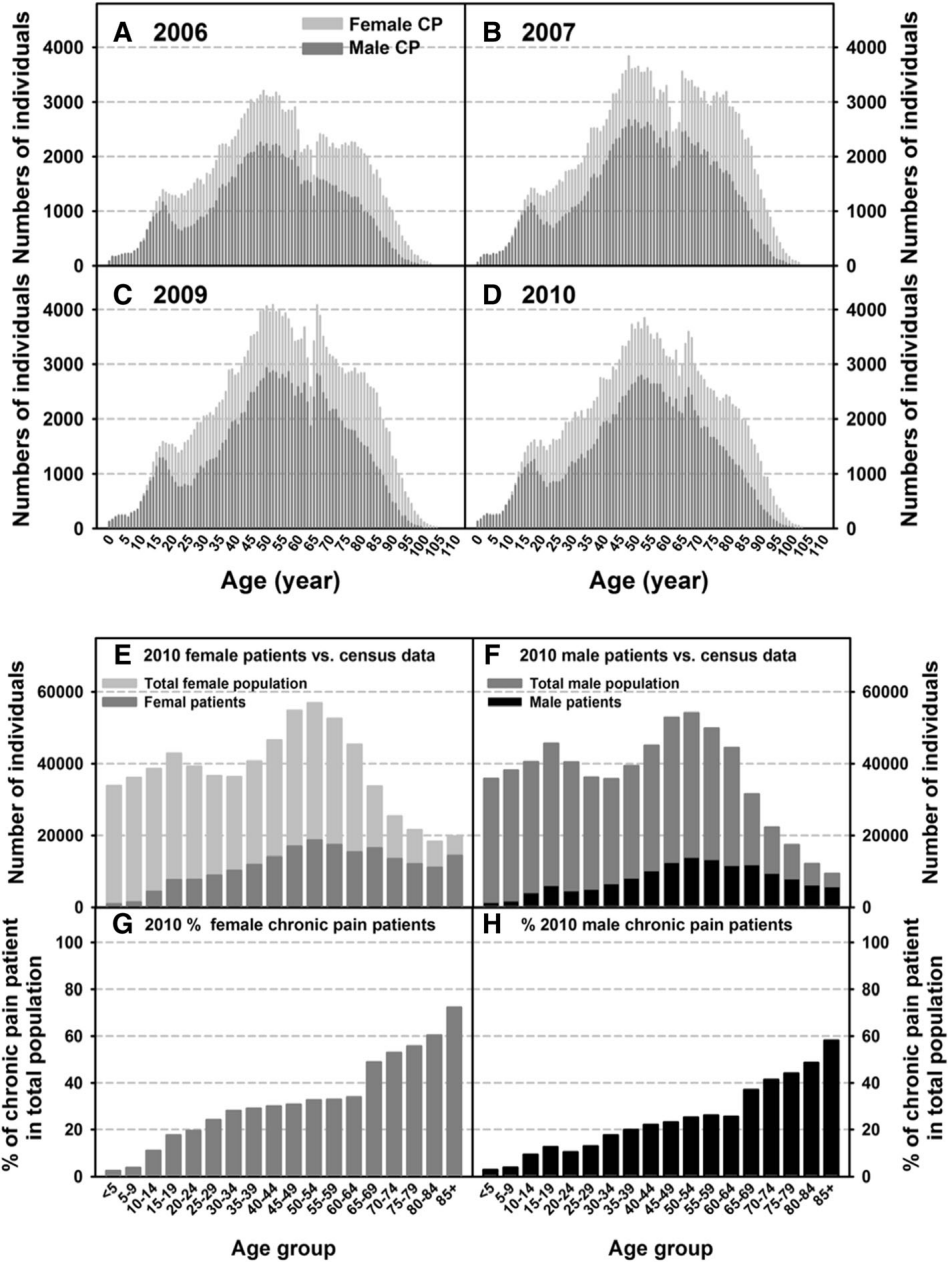
Age and gender distribution of the chronic pain cohort. Distribution of male and female chronic pain patients by age are graphed for each calendar year: 2006 (**a**), 2007 (**b**), 2009 (**c**), and 2010 (**d**). Genders and ages, as reported in five-year increments, of the chronic pain cohort were compared to the total Maine population, as reported by the 2010 Maine Census: females (**e**) and males (**f**). The percent of chronic pain patients for each gender and age group within the perspective Maine population were used to indicate the prevalence of chronic pain in Maine: female (**g**) and male (**h**)

All chronic pain patients identified from the MEAPCD are grouped based on individual ages and separated by gender (Figs. 3a–h). Across all four years, the numbers of female chronic pain sufferers are higher compared to males in all age groups, and the prevalence of chronic pain peaked around 50–55 years of age for both males and females (Figs. 3a-d). To compare the age distribution of chronic pain patients to the total Maine population in 2010, the chronic pain cohort was graphed against the total Maine population, as reported in the US Census (Fig. 3e and f), which was further analyzed as a percentage of the total Maine male and female population respectively within each age group in 2010 (Figs. 3g and h). The 2010 chronic pain prevalence is significantly higher in females than in males (Figs. 3g and h; 3g vs. 3h *p* < 0.001 at the overall prevalence (in %) and each corresponding age group with the exception of the < 5 and 5–9 age groups). It is also clear that the percentage of the chronic pain population increases with age, with a sharp increase at age 65 and above in both males and females. However, the chronic pain population of females reached 20% of the total Maine female population between the ages 20–24, whereas males do not reach 20% until the ages 35–49; the female chronic pain population was at 50% of the total Maine female population between the ages 70–74, compared to the male chronic pain population that reached 50% at 85 years plus (Figs. 3g and h).

### Chronic pain cases by Maine counties

**Fig. 4:**
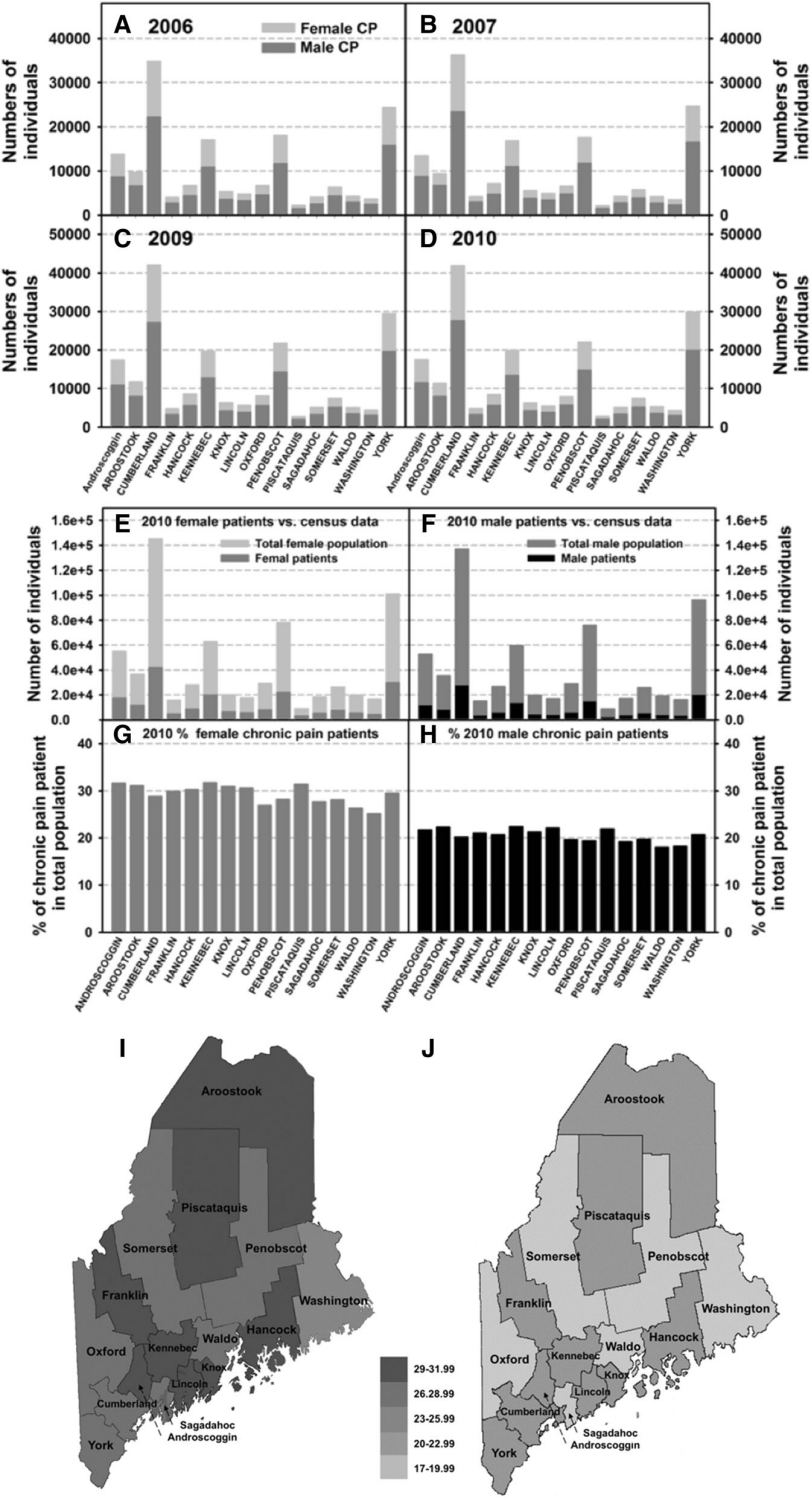
Chronic pain cases by county. The total numbers of male and female chronic pain patients per Maine county are shown for the following calendar years: 2006 (**a**), 2007 (**b**), 2009 (**c**), and 2010 (**d**). County of residence are classified as county of residence during the calendar year of reporting. The numbers of female (**e**) and male (**f**) chronic pain patients per county were compared to the total numbers of females and males living in that county in 2010, as reported by the 2010 Maine Census. The percent of chronic pain patients within the perspective Maine population were used to indicate the prevalence of chronic pain in each of the Maine county: female (**g**) and male (**h**). These same percentages of the female (**i**) and male (**j**) chronic pain cohort within the population of each Maine county are also shown on the state map. Percentages are presented in a density scale and the same scale is used in Figs. **i** and **j**

County distribution of chronic pain patients was reported based on the county of residence at the time they were identified in the MEAPCD (Fig. 4a–d). As expected, there were higher numbers of chronic pain patients in counties that had overall greater total populations. The four counties with the greatest chronic pain patients were Cumberland, York, Penobscot, and Kennebec. Similar to what we saw with age groups, the numbers of female chronic pain patients were greater than male patients in all counties. The number of chronic pain patients in 2010 were further graphed against the total population within each county (Figs. 4e and f) [24]. The prevalence of chronic pain was also significantly higher in females than in males across all counties (Figs. 4g–j; 4g vs. 4h comparison within each county, *p* < 0.001). Specifically, percentages of female chronic pain patients were above 25% in all counties (Fig. 4g), while none of the counties had a percentage of male chronic pain patients greater than 25% (Fig. 4h). It should be noted that counties that had greater numbers of chronic pain patients are not always the same counties that had the higher percent of chronic pain patients (e.g., Cumberland County vs. Piscataquis County).

### Types of healthcare insurance by chronic pain patients

**Fig. 5:**
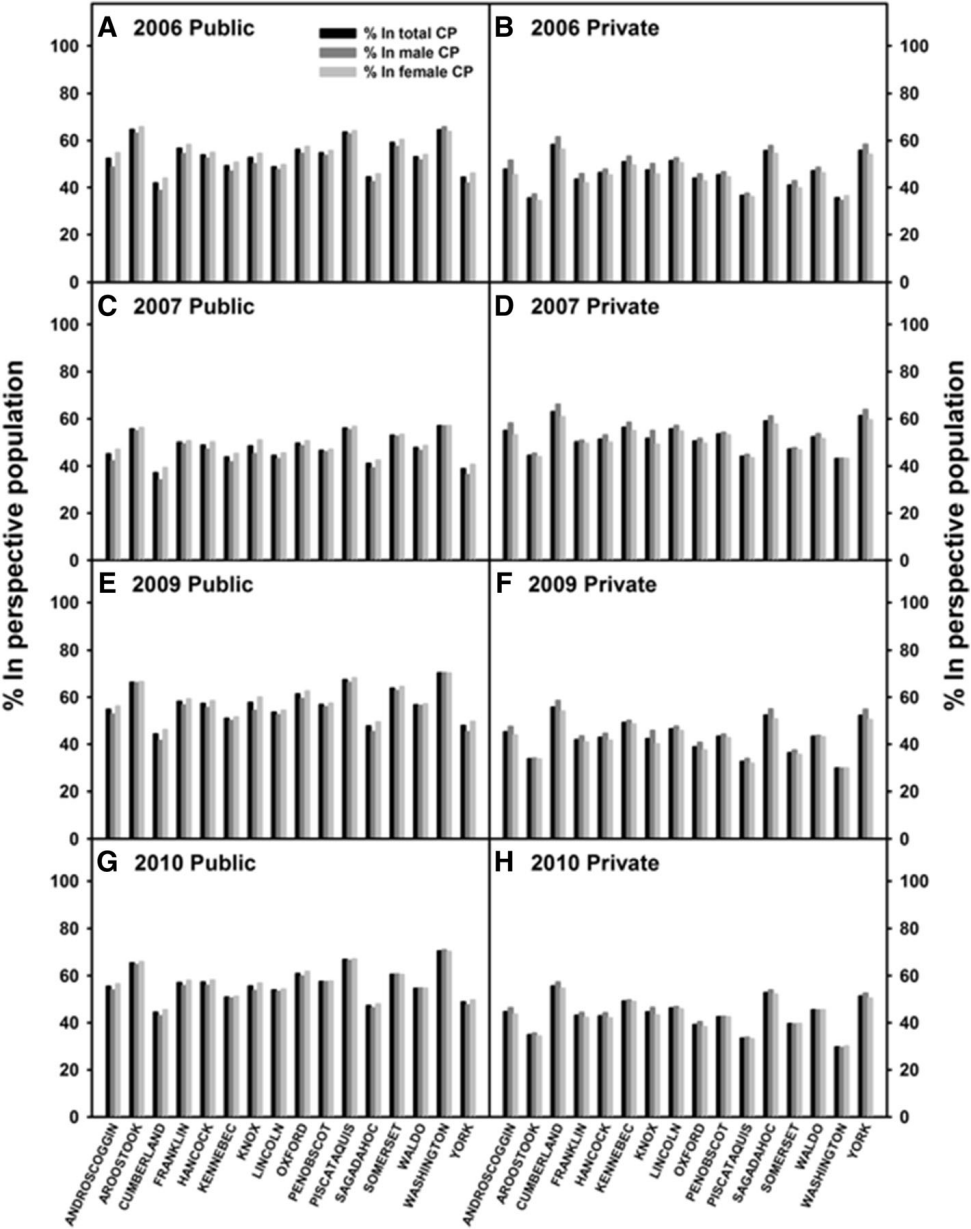
Estimates of healthcare insurance types in the perspective chronic pain population across the state. Distribution of chronic pain patients who receive public (**a**, **c**, **e**, and **g**) versus private (**b**, **d**, **f**, and **h**) primary insurances are shown for each Maine county. County of residence is classified as county of residence during the calendar year of reporting. Graph is displayed as follows: 2006 public healthcare recipients versus (**a**) 2006 private healthcare users (**b**), 2007 public healthcare recipients versus (**c**) 2007 public healthcare users (**d**), 2009 public healthcare recipients versus (**e**), 2009 public healthcare users (**f**), 2010 public healthcare users versus (**g**) 2010 public healthcare users (**h**)

MEAPCD classifies insurance types for each person as public or private based on the type of insurance used to pay the claim**;** therefore, a state-wide distribution of chronic pain patients receiving public vs. private insurance was determined. The percentage of patients (within the perspective chronic pain populations) who used public (Figs. 5a, c, e, and g) and private (Figs. 5b, d, f, and h) health insurance were reported based on individuals’ gender and county of residence during the indicated calendar year. No differences in insurance utilization by gender were observed. Among all counties, based on average insurance usage in the total chronic pain population, a significantly higher percent of chronic pain patients used public insurance than private insurance in 2006 (public 53.7% vs. private 46.3%), 2009 (public 57.1% vs. private 42.9%) and 2010 (public 56.6% vs. private 43.4%) (*p* < 0.001 for all), while in 2007, more chronic pain patient utilized private insurance than public insurance (public 47.7% vs. private 52.3%) (*p* < 0.001 for all). These data suggest a potential burden that chronic pain is placing on the state’s public healthcare system, which highlights the importance of earlier management and preventative care for chronic pain patients.

### Relationships between county-wide demographics and chronic pain prevalence

**Fig. 6:**
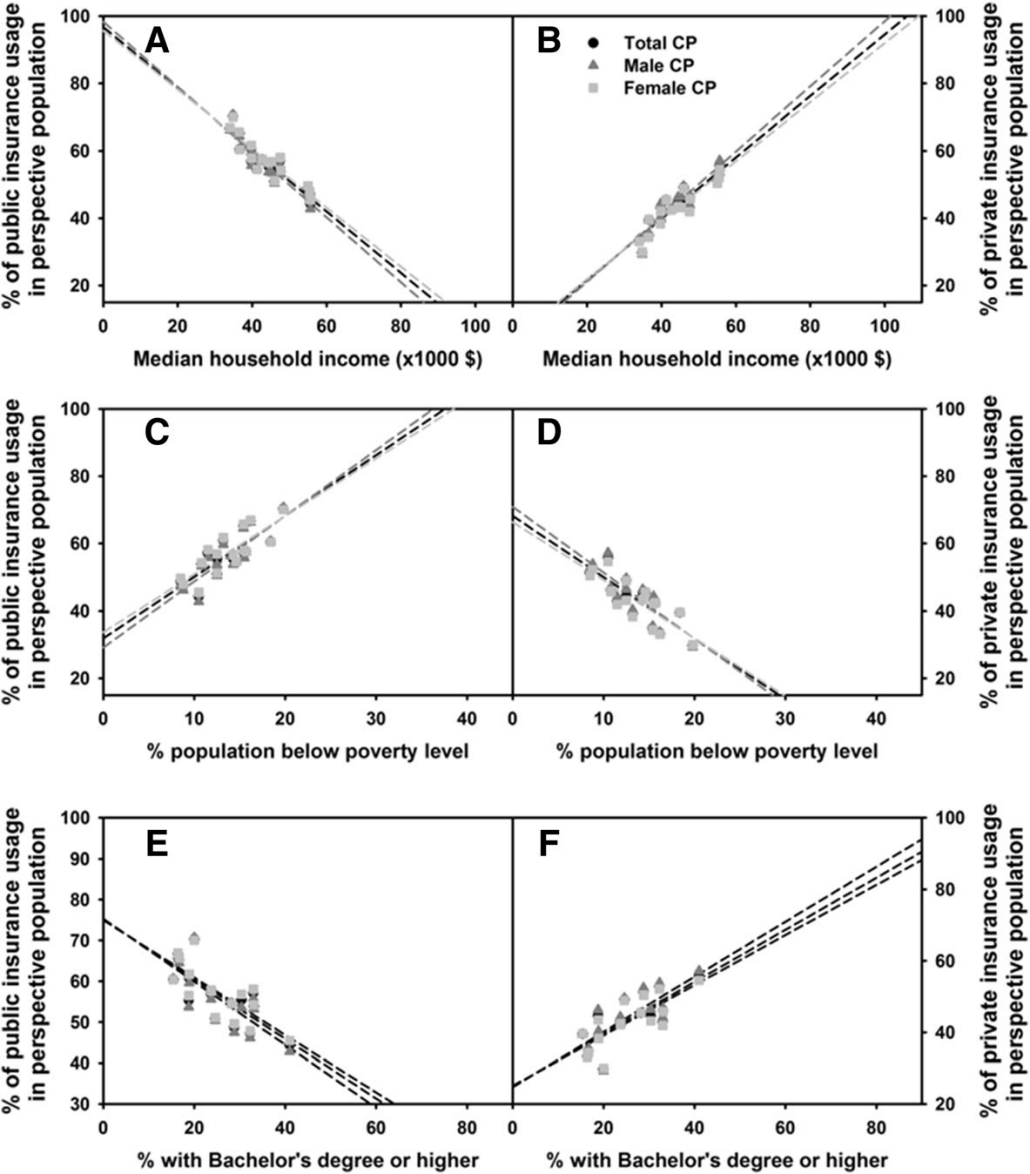
Association between socioeconomic factors and the usage of public health insurance. The percentages of individuals who used primarily public (**a**, **c** and **e**) and private (**b**, **d**, and **f**) insurances were compared to the median household income (**a** and **b**), percent population below poverty level (**c** and **d**), and percent with bachelor’s degree or higher (**e** and **f**) using the data from the 2010 chronic pain cohort and 2010 Maine Census

Health disparities that are common to chronic pain sufferers have not been fully identified and this is one of the goals of the National Pain Strategy. In an attempt to shed light on this area, the relationships between the percent of Maine’s chronic pain population (males vs. females vs. total) and socioeconomic factors were examined at county level. These factors included: (1) median household income, (2) percentages of the population living below the poverty line, and (3) educational status (percentages of the population who had a bachelor’s degree or higher/ percent of people who completed high school). Data on median household income, poverty level, and education status within county were obtained from the 2010 Maine Census. Surprisingly, no correlations were detected with any of the factors examined (data not shown). However, when we examined the relationships between these factors and the types of insurance used, significant correlations were observed (Figs. 6a–f, *p* < 0.001 for all correlations). Specifically, lower median household income, a higher percentage of the population living below the poverty line, and a lower percentage of the population who had a bachelor’s degree or higher, are all positively correlated with the percent of chronic pain patients who used the public insurance, while negatively correlated with the percent of chronic pain patients who used the private insurance.

## Discussion

Maine is currently one of 30 states in the US that maintains an APCD (All Payer Claims Database, a collection of insurance claim data), is in the process of implementing an APCD, or has strong interest in implementing an APCD [32]. APCDs are a relatively new resource for health data that can be utilized for large scale population studies. The current study used MEACPD to identify and examine the chronic pain population in the state, with the hope to recognize areas for improvement and populations that are experiencing the greatest rates of chronic pain [33, 34]. In turn, we hope that this information will be used to design strategies that address possible health disparities associated with the chronic pain sufferers.

According to others’ reports, at any given point, 30% of the adult population suffers from chronic pain [1, 35]. We used a previously validated method to identify the chronic pain population from the MEAPCD [28]. On average, our parameters identified 330,054 people from a total of 1,119,509 individuals, or 29.5% of the total Maine population (range 25–30%) as having chronic pain, which is similar to the reported percentages. Since we did not include people with ICD-9 codes associated with facial or migraine pain, and the database did not include people without insurance or individuals utilizing veterans’ assistance programs, the actual numbers of chronic pain sufferers in the state are likely to be higher.

Maine was one of 30 states that saw a significant increase in opioid-related deaths between 2010 and 2015, increasing from 9/100,000 opioid-related deaths in 2010 to 23/100,000 deaths in 2015 [36]. Trends in nation-wide opioid-related deaths have been on the rise since 2000 [12], which corresponds to the quadrupled prescribing rates of opioids as a pain reliever seen between 1999 and 2008 [37]. Consistent with these findings, we did notice a gradual increase in the population that had an opioid prescription for 90 consecutive days or more (43,846 (15.8%) in 2006; 56,498 (16.5%) in 2007; 59,985 (16.5%) in 2009; and 63,475 (18.8%) in 2010). Many have speculated the opioid use for chronic pain is partially to blame for the current epidemic. Contrary to this, in our study the majority of the chronic pain cohort was identified through the ICD-9 code criteria (> 80%) and not through the opioid prescription criteria. We found only 15.8–18.8% of chronic pain patients received an opioid prescription for more than 90 consecutive days (Fig. 2), which is consistent with the observation made by Tian et al. (2013), which found that 17% of a chronic pain population received at least a 90-day prescription of opioids. However, it was noted by Tian et al. that many chronic pain patients received opioids for less than 90 consecutive days as 43% of the chronic pain population receiving any opioid prescription over the course of a year [28]. It is important to mention that opioid prescriptions do not indicate if the prescriptions were filled or used as directed. In future studies, it would be very valuable to determine whether the reason for many chronic pain patients not receiving long-term prescription of opioids is that they received other types of pain treatment or that they simply did not have access to adequate pain management.

All age ranges were included for this study because: 1) the median age of the state of Maine is greater than most other states, and 2) chronic pain conditions in populations under the age of 18 have been found to be increasing over the past few decades [38], emphasizing the importance of monitoring and treating chronic pain in children. Our data showed that the percent of chronic pain patients increased exponentially with age, particularly after age 65 (Fig. 3g and h). This is supported by other studies that reported increased prevalence of chronic pain in older populations [35, 39–41]. As Maine is one of the oldest states in the nation, Maine faces a potentially greater burden of chronic pain. Studies have found that people over the age of 35 are at increased risk for developing chronic pain conditions [26, 27, 29, 42]. Our study revealed that the greatest number of chronic pain sufferers were between the ages of 50 and 55 in both males and females (Figs. 3a–3f). This is also of relevance to the state’s economy as Maine has more 50–55 year-olds in the work force due to the median age of the state being higher than national average. With chronic pain increasing in this age group, there are more individuals who may find working difficult with chronic pain and more likely to require government assistance (Fig. 6). Thus, ensuring adequate management and prevention of chronic pain are particularly crucial for the Maine economy.

One of the most notable findings from our data analysis is that the prevalence of chronic pain was significantly higher in females than in males in all Maine counties, and in all age groups, with the exception of individuals younger than 10 (Figs. 3 and 4). Previous studies have observed greater prevalence of chronic pain in females compared to males [26, 43]. This may indicate the increased length of chronic pain experienced by women, thus one explanation for why the prevalence of chronic pain remains higher in women compared to men nationwide. The exact reason for why chronic pain is more prevalent in women is not fully understood. It may be related to the fact that women tend to use healthcare more frequently than men [27], and that sex-linked differences in the neurobiology of pain and pain perception. Furthermore, Maine women earn less than men and are more likely to live in poverty, which can put them at greater risk for developing chronic conditions [44, 45].

Employment status, occupational factors, education, and income have been inversely associated with chronic pain [46–49]*.* Our study did not detect an association between the prevalence of chronic pain vs. levels of education or income (data not shown) based on Maine county reports. In 2010, Maine had an education level higher than the national average [25, 50], which may explain the lack of correlation between the prevalence of chronic pain and education status. Also*, s*ince all data are analyzed at the county level, our study may not be sensitive enough to detect these correlations because Maine counties are composed of more towns than cities and the diversity between the two may mask any differences that may be seen at the individual level*.* However, we did reveal significant negative correlations between income and education levels and the usage of public insurance (Fig. 6), highlighting the close relationship between one’s socioeconomic status and health insurance type. Future detailed analysis at individual levels is necessary to reveal the relationships between the prevalence of chronic pain and socioeconomic status, education status, or usage of the public health system within the state of Maine.

Comparing the insurance used by chronic pain patients allowed us to determine if there is an association between chronic pain prevalence and the insurance type (private vs. public assistance) utilized by chronic pain patients. In 2009 and 2010, more of the chronic pain cohort used publicly funded healthcare. In 2009, Maine residents utilized Medicaid at the third highest rate in the country [51]. This increase indicates that a need for government assistance corresponds with the economic crash of 2008, which resulted in an increased number of Maine residents filing for unemployment; possibly exacerbating any chronic pain conditions, as it is known that distress experienced by unemployment, regardless of education, only increases likelihood of chronic pain development and the duration of the episode [4, 7, 9, 27, 40, 52]*.* Furthermore, the longer a person is out of work from chronic pain, the less likely they are to return back to work [53]. Tian et al. (2013), also observed that more chronic pain patients were receiving Medicaid benefits than non-chronic pain patients within the same treatment facility [28]. In addition, Maine has higher usage rates of public assistance compared to other states. Thus, these findings emphasize the importance of addressing the issue of chronic pain in the state, because chronic pain conditions could lead to more healthcare-associated costs, decrease a person’s ability to work, and increase the need for government assistance due to this loss of work.

## Conclusions

To our knowledge, this is the first study identifying and examining the prevalence of chronic pain in the state of Maine. Our results indicate a significantly higher prevalence of chronic pain in females than in males at almost all age groups and in all Maine counties. The burden that chronic pain exerts on the public health system is related to patients’ socioeconomic status and educational level. Strategies at the state levels should be developed to combat this serious health problem.

## Additional file

Additional file 1: Opioid drugs that were prescribed by providers in the state of Maine during the selected study time period (2006-2011). (DOCX 21 kb)

## Abbreviations

APCD: All Payer Claims Database
MEAPCD: The Maine All Payer Claims Database
PPV: Positive Predictive Value (PPV)
