# Supplementary material for: Characterizing the demographics of chronic pain patients in the state of Maine using the Maine all payer claims database
Source: BMC Public Health. 2018 Jun 28;18:810. doi: 10.1186/s12889-018-5673-5 (PMC6022454; doi:10.1186/s12889-018-5673-5)
Supplement: Supplementary file 1 — Opioid drugs that were prescribed by providers in the state of Maine during the selected study time period (2006-2011). (DOCX 21 kb) [file 12889_2018_5673_MOESM1_ESM.docx]

**Additional file 1**

Opioid drugs that were prescribed by providers in the state of Maine during the selected study time period (2006-2011).

ACTIQ

ACTIQ

ACTIQ 1,20

ACTIQ 200M

ACTIQ 800

ACTIQ LOZ

ANEXSIA

APAP-CODEI

APAP/CODEI

APAP/TRAMA

ASA/CODEIN

ASCOMP

ASCOMP CAP

ASCOMP W/C

ASCOMP WIT

ASCOMP/COD

ASPIRIN W/

ASPIRIN WI

ASPIRIN-CO

ASPIRIN/CO

ATUSS G

ATUSS HC

ATUSS HD

ATUSS HS

ATUSS HS S

ATUSS HX

ATUSS MR

ATUSS MS

ATUSS NX

AVINZA

AVINZA

AVINZA 1 1

AVINZA 1 3

AVINZA 1 4

AVINZA 1 6

AVINZA 1 7

AVINZA 1 9

AVINZA 120

AVINZA 30

AVINZA 30M

AVINZA 45

AVINZA 60

AVINZA 60M

AVINZA 75

AVINZA 90

AVINZA 90M

AVINZA CAP

B-TUSS

B-TUSS

BALACET 32

BELLA/OPIU

BIOTUSSIN

BROMCOMP H

BRONTEX

BUPRENEX

BUPRENEX

BUPRENEX I

BUPRENORPH

BUTORPHANO

BUTRANS

BUTRANS 1

BUTRANS 10

BUTRANS 20

BUTRANS 5

CAPITAL AN

CAPITAL W-

CAPITAL W/

CAPITAL WI

CAPITAL/CO

CHERATUSSI

CO-GESIC

COCET

CODAL-DH

CODAL-DH S

CODAMINE

CODEINE 10

CODEINE PH

CODEINE SU

CODEINE-GG

CODEINE-GU

CODEINE/AC

CODEINE/GG

CODEINE/GU

CODEINESUL

CODICLEAR

COMBUNOX

COMBUNOX T

COMPOUND

COMPOUND P

COTUSS-V

COTUSS-V S

CYTUSS HC

CYTUSS-HC

DAMASON-P

DARVOCET

DARVOCET

DARVOCET A

DARVOCET T

DARVOCET-N

DARVON

DARVON

DARVON 65

DARVON CAP

DARVON COM

DARVON-N

DARVON-N 1

DARVON-N T

DAYPRO TAB

DETUSS

DIHYDRO-GP

DIHYDRO-PE

DIHYDROCOD

DILAUDID

DILAUDID

DILAUDID 1

DILAUDID 2

DILAUDID 4

DILAUDID 8

DILAUDID I

DILAUDID S

DILAUDID T

DILAUDID-5

DILAUDID-H

DISKETS

DISKETS TA

DOLOPHINE

DOLOPHINE

DRITUSS HD

DROTUSS-CP

DURAGESIC

DURAGESIC

DURAGESIC-

DYNATUSS H

EMBEDA

EMBEDA 1 1

EMBEDA 20-

EMBEDA 30-

EMBEDA 50-

EMBEDA 60-

EMBEDA CAP

ENDOCET

ENDOCET

ENDOCET 1

ENDOCET 10

ENDOCET 5-

ENDOCET 5/

ENDOCET 7.

ENDOCET TA

ENDODAN

ENDODAN 1

ENDODAN 4.

ENDODAN TA

ETH-OXYDOS

EXALGO

EXALGO

EXALGO 1 8

EXALGO ER

EXALGO TAB

FENTANYL

FENTANYL

FENTANYL 1

FENTANYL 2

FENTANYL 5

FENTANYL 7

FENTANYL C

FENTANYL D

FENTANYL O

FENTANYL T

FENTORA

FENTORA

FENTORA 10

FENTORA 20

FENTORA 40

FENTORA 60

FENTORA TA

FENTUSS EX

FIORICET W

FIORICET/C

FIORINAL C

FIORINAL W

FIORINAL/C

FIORTAL/CO

GG/CODEINE

GIANVI

GUAIFEN-C

GUAIFEN-C

H C TUSSIV

H-C TUSSIV

HC TUSSIVE

HC-GUAI

HC-PE-DBRO

HC/GUAI

HC/PE/DBRO

HISTINEX H

HISTINEX P

HISTUSSIN

HYCET

HYCET 7.5

HYCET SOL

HYCET SOLU

HYCODAN

HYCOMINE C

HYCOTUSS

HYCOTUSS E

HYD POLST-

HYDRO-DP

HYDRO-TUSS

HYDROCO-AP

HYDROCO/AP

HYDROCO/IB

HYDROCOD P

HYDROCOD-H

HYDROCOD/G

HYDROCOD/H

HYDROCOD/I

HYDROCODON

HYDROFED

HYDROGESIC

HYDROMORPH

HYDRON CP

HYDRON EX

HYDRON KGS

HYDRON PSC

HYRDROCODO

IBUDONE

INFUMORPH

KADIAN

KADIAN

KADIAN 1 1

KADIAN 1 2

KADIAN 1 3

KADIAN 1 5

KADIAN 1 6

KADIAN 1 8

KADIAN 10

KADIAN 100

KADIAN 20

KADIAN 200

KADIAN 20M

KADIAN 30

KADIAN 50

KADIAN 60

KADIAN 80

KADIAN CAP

KADIAN ER

KADIAN SR

LEXUSS 210

LORCET

LORCET 10-

LORCET 10/

LORCET PLU

LORCET TAB

LORTAB

LORTAB 10

LORTAB 10

LORTAB 2.5

LORTAB 5

LORTAB 5 T

LORTAB 7.5

LORTUSS HC

M-OXY

MAXI-TUSS

MAXIDONE

MAXIDONE 1

MAXIDONE T

MEDTUSS HD

MEPERIDINE

MEPERITAB

MEPERITAB

MEPROZINE

MEPROZINE

METHACONE

METHADONE

METHADONEH

METHADOSE

METHADOSE

MOPRHINE S

MORPH SULF

MORPHINE

MORPHINE

MORPHINE 1

MORPHINE 2

MORPHINE 4

MORPHINE 5

MORPHINE 8

MORPHINE S

MORPHINE-N

MORPHINESU

MS CONTIN

MS CONTIN

MSIR

MSIR

MSIR 30 MG

MSIR SOL 2

MSIR TAB 1

MSIR TAB 3

Name

NASOTUSS

NORCO

NORCO 7.5-

NORCO TAB

NOTUSS AC

NUBAIN

NUBAIN

NUBAIN INJ

NUCYNTA

NUCYNTA

NUCYNTA 1

NUCYNTA 10

NUCYNTA 50

NUCYNTA 75

NUCYNTA ER

NUCYNTA TA

OPANA

OPANA

OPANA 1 10

OPANA 1 5

OPANA 10 M

OPANA 5 MG

OPANA ER

OPANA ER

OPANA ER 1

OPANA ER 2

OPANA ER 3

OPANA ER 4

OPANA ER 5

OPANA ER T

OPANA TAB

OPANAER

OPIUM

OPIUM TIN

OPIUM TINC

ORAMORPH S

ORAMORPHSR

OXYCO/APAP

OXYCOD-APA

OXYCOD/APA

OXYCOD/ASA

OXYCOD/IBU

OXYCODO-AP

OXYCODON

OXYCODON H

OXYCODON-A

OXYCODONE

OXYCODONE-

OXYCODONE/

OXYCODONEA

OXYCODONEH

OXYCONTIN

OXYCONTIN

OXYDOSE

OXYDOSE 20

OXYDOSE CO

OXYFAST

OXYFAST CO

OXYIR

OXYIR 5 MG

OXYIR CAP

OXYMORPHON

PALLADONE

PANCOF EXP

PANCOF PD

PANCOF XP

PAREGORIC

PAREGORIC

PENTA/APAP

PENTAZ/NAL

PENTAZOCIN

PERCOCET

PERCOCET

PERCOCET 1

PERCOCET 2

PERCOCET 5

PERCOCET 7

PERCOCET T

PERCODAN

PERCODAN T

PERCOLONE

PERCOLONE

PHENDACOF

PRO-COF

PRO-COF SO

PROMETH/CO

PROPACET 1

PROPO-N/AP

PROPOXACET

PROPOXY HC

PROPOXY-N/

PROPOXY/AP

PROPOXYPH-

PROPOXYPHE

Q-V TUSSIN

REPREXAIN

RESPA A.R.

RESPA C&C

RESPA DM

RESPA DM

RESPA-1ST

RESPA-A.R.

RESPA-BR

RESPA-DM

RESPA-PE

REZIRA

RINDAL HPD

ROXANOL

ROXANOL

ROXANOL 10

ROXANOL 20

ROXANOL SO

ROXANOL-T

ROXICET

ROXICET

ROXICET 1

ROXICET 2

ROXICET 5-

ROXICET 5/

ROXICET SO

ROXICET TA

ROXICODONE

ROXILOX

RYDEX

RYZOLT

RYZOLT ER

RYZOLT TAB

STADOL

STADOL NS

STAGESIC

STAGESIC 5

SUBOXONE

SUBOXONE

SUBOXONE 1

SUBOXONE 2

SUBOXONE 8

SUBOXONE M

SUBOXONE S

SUBOXONE T

SUBUTEX

SUBUTEX

SUBUTEX 1

SUBUTEX 2

SUBUTEX 8

SUBUTEX 8M

SUBUTEX SU

SYNALGOS

SYNALGOS D

SYNALGOS-D

TALACEN

TALACEN TA

TALWIN NX

TAPENTADOL

TRAMADL/AP

TRAMADO/AP

TRAMADOL

TRAMADOL

TRAMADOL H

TRAMADOL-A

TRAMADOLHC

TRAMADOLHY

TUSSEND

TUSSEND EX

TUSSICAPS

TUSSIGON

TUSSIGON T

TUSSIONEX

TUSSIONEX

TUSSIONEXP

TYLENOL W/

TYLENOL WI

TYLENOL-CO

TYLENOL/CO

TYLOX

TYLOX 5-50

TYLOX 5/50

TYLOX CAP

ULTRACET

ULTRACET

ULTRACET T

ULTRAM

ULTRAM

ULTRAM 1 5

ULTRAM 50

ULTRAM 50M

ULTRAM ER

ULTRAM ER

ULTRAM TAB

VANACET 5/

VAZOTUSS H

VI-Q-TUSS

VI-Q-TUSS

VICOCLEAR

VICODIN

VICODIN

VICODIN 5-

VICODIN 5/

VICODIN ES

VICODIN HP

VICODIN TA

VICODIN TU

VICOPROFEN

VORTEX

VORTEX HOL

VORTEX VAL

VORTEX/MAS

WELLTUSS E

XODOL

XODOL

XODOL 10-3

XODOL 10/3

Z-TUSS AC

Z-TUSS DM

ZYDONE

ZYDONE 10-

ZYDONE 10/

ZYDONE 5/4

ZYDONE 7.5

ZYDONE TAB
